# Aids to Nursing

**Published:** 1888-02-25

**Authors:** M. M. Basil

**Affiliations:** Author of "The Commoner Accidents to Life and Limb"


*(To be continued.)*

### Aids to Nursing.

By M. M. Basil, M.A., M.B., C.M., Author of "The Commoner Accidents to Life and Limb."

*(Continued from p. 348.)*

In connexion with the hygiene of the sick-room, we must take a rapid review of some of the requirements of healthy habitations, in so far as they are likely to come under the control of the nurse. An elementary knowledge on this point must be imparted to you here, as your attention may not be called to the subject during your practical training. In a hospital the sanitary arrangements are looked after from time to time, and nearly all responsibility on this score is taken off your shoulders: in private and district nursing a good deal will depend upon you. An intelligent nurse with a clear knowledge of the subject, having opportunities of seeing almost every portion of a house, is in a much better position to see its sanitary defects than the visiting physician. Perhaps some of you do not intend to make nursing the business of your life. For such there is no better lesson to be got during their training than a knowledge of the requirements of healthy living. Then again, whether you like it or not, you will come to be looked upon in after-years as authorities, on a small scale, on matters concerning health and its derangements. You should therefore have some clear notions on the subject. Not only every nurse, but every woman, should have some elementary knowledge of sanitation, because in reality most of our fevers and preventible diseases are processes of poisoning arising from want of knowledge in the person, who of all others in the house, is in the best position to prevent such accidents. We think it pious to call them diseases and dispensations of Providence. Curiously enough, the dispensations figure largely as the peculiar heritages of the poor and the ignorant.

The sick-room and its surroundings ought to be clean in the highest degree. All matter in the wrong place is dirt. There are various degrees of the nuisance, but uncleanliness and untidiness of what is in sight and out of sight are the harbours and sources of dirt. Cleanliness in medicine and surgery is not next to godliness but far above it. To be efficient, it must be a living faith manifesting itself constantly in works. Have no hypocrisy in soap and water; no sham scrubbing can make your sick-room healthy. No room or passage in the house should smell offensively. No room is clean that can be made cleaner, and no place is healthy that emits offensive smells. Aim to keep what is out of sight clean, and then you may be sure what is in sight will take care of itself. If we must have dirt, let us, as the Quaker said, have it in the middle of the room, where we can see it. Lockers, if in use, must be kept open and perfectly clean. If left entirely under the patient's control, they will become nests of abomination and nastiness. See that no decomposing rubbish is kept under the bedding or bedstead. Valances are apt to become screens for concealing magazines of offensive material. Do not place basins containing lotions or other fluids under the bedstead or under tables where they cannot be seen and may be upset, or in positions where they may cause any one to stumble.

Especial attention must be paid to the cleanliness of every place that is connected with the sewage-pipes. If the drainage is found out of order in a private house, it is your duty to report the fact privately to the doctor in attendance. If the basement of the house smells offensively, if damp is seen rising up the walls, and if rats have been seen frequently in the cellars, then the drainage is likely to be highly defective. Baths, fixed lavatory basins, w.c.'s, kitchen and scullery traps, grease traps, the pantry and the cellars, must receive your particular attention. Always see personally to the cleanliness of the w.c. at least twice daily. Flush it from time to time. The flushing should stop *before* or *as soon as* the cistern is empty. A continuous slow flow of water is useless, if not actually dangerous. Windows in sinks and w.c.'s should be left open, top and bottom every day. No towels, socks, rags, etc., should be allowed to enter the soil-pipes. Cellars and lumber-rooms must be well ventilated, as the air from them is certain to rise up by means of corridors and stairs, and also by diffusion through walls, into every room in the house. No infectious case should ever be kept in the basement floor of any house, especially when the sick are crowded together. The absurdly original idea of having a dissection-room in the basement of a hospital is one that could only occur in the high state of civilization peculiar to London.

What is still known in some quarters as tidying or dusting of rooms is a process that no woman with pretensions to any intelligence should follow. The mechanical raising of dust by a fly-flapping action simply displaces the dust from one place to several other positions. Wiping with a damp cloth is the method to be recommended. Wash and scrub no floors in the sick-room if possible; never do so in the afternoon, as the room will be damp through the night. Get the patients out of the room until the floor is perfectly dry. Foot-mats and carpets, if in use, must not be laid down until the room is dry. The floors of a ward should be rendered capable of dry rubbing and polishing, and frequent washing of them should be avoided. Dr. Langstaff, of Southampton, strongly recommends solid paraffin for this object. "The paraffin is melted and then poured on the floor, and ironed into it with a box-iron, heated from the interior by burning charcoal; it penetrates about a quarter of an inch into the wood. The excess of paraffin is scraped off, and the floor is brushed with a hard brush; a little paraffin in turpentine is then put on, and the flooring is good for years. An experience of some years in Southampton Infirmary has proved the advantage of this flooring. It has also been introduced with satisfactory results into the Bristol Infirmary." "The practice of frequently washing the floors in hospitals," says Dr. Parkes, "is well known to increase the chance of erysipelas (and kindred diseases), and this might be explained, as Von Nägeli suggests, by the moisture and subsequent drying helping the development and subsequent dissemination of minute organisms."

The hands and face of the patient ought to be washed regularly. In no case should you use the same water for washing two or more patients, as dangerous diseases may thus be communicated. If the same basin is to be used for several patients, scald it out, and use a disinfectant in all cases of doubt, and certainly in every case of eye disease coming under your care. The patient's teeth ought to be brushed or wiped perfectly clean at least every morning. Every patient should have a separate towel. This rule is absolute in cases of eye affections or of eruptive diseases. A similar statement holds good regarding tooth-brushes, razors, etc. If there is any deficiency in the supply of towels, sheets, brushes, basins, or dressings, a report to that effect should be sent to head-quarters through the proper channels.

Some patients are said to have a mania for washing: when such a condition does exist outside an asylum, it is, of all forms of insanity, the one that requires least interference. Only, it is a pity we do not know any method of spreading it as an epidemic among shoals of the dirty sane. If there is one superstition that is useful, it is superstition on matters of scrupulous cleanliness. When possible, every patient ought to get a warm bath at least once a week. The feet should be washed regularly twice a week. No nurse should be a strict economist in soap and water, especially as most hospital patients are yet terribly ignorant of these tests of civilisation. To keep infection out of your wards, you must attend at once to offensively smelling feet, to scabies, tinea, etc.

In women and children, and sometimes also in men, the hair must be carefully inspected during long confinement to bed. Misery acquaints a man with strange bed-fellows, and to your astonishment you may find creeping, jumping, and other kindred cattle, congregated in shoals and nations in situations where you least expected them. There is really no excuse for negligence in this direction.

Materials used for dressing wounds, for making poultices for abscesses, etc., should be burnt. In the long run it is the most economic course. In district nursing, and especially in the lying-in condition, in place of macintosh, papier goudronne may be used with advantage. The paper costs very little and can be burnt after use. In certain quarters there is a tendency to perilously short-sighted economy in matters of dressing, splints, and other surgical appliances. Such thrift is literally dreadful. In many books on nursing there is very dangerous teaching on this subject, based, no doubt, on the laudable desire to prevent waste. Miss Lückes, for one, takes much pains to explain the method of cleaning wooden splints that have been used in cases of gangrene, erysipelas, pyæmia, etc. The only safe means to render such a splint harmless is to put it into the fire. If hospitals are to be looked upon as "houses of charity," intended to benefit the sick, such a course is not only the safest, but the most economical. A small item in one department of the hospital expenses must not be kept down at the terrible risk of sending up very unduly half-a-dozen other items. In the surgical wards especially, it is well to remember that however well-intentioned and attentive we may be, we may do great harm to the patients unless we conduct everything persistently and intelligently on the strictest principles of radical cleanliness. Every person working in a sick ward would do well to remember the prayer offered by the Scotch minister for the General Assembly: "May the Assembly be guided as not to do harm." Our hands, dressings, splints, hospital wards, cannot be kept too free from sources of grave risks to our patients. Judging from even very recent expressions of opinion on the subject, it would appear that in the matter of surgical cleanliness the older and more experienced nurses are the ones most likely to be found deficient. "The most experienced nurse," says a recent writer, who shall not be named here, "has to be most mistrusted." Another writer sees salvation only in "fluffy hands," as an evidence that the nurse has used the proper strength of carbolic acid lotion efficiently: a poor broken reed or rubbishy fluff to depend upon, if the hands are not under the control of an intelligent head, fully conscientious and aware of the extent of responsibilities that are incurred! It is well to remember that the tendency of mere mechanical experience is to make one negligent of attending to apparently non-essential details in the sick-room, as otherwise our service to the sick will be an unmixed evil.

Patients with large wounds, especially during the first three or four days after their infliction, are particularly apt to suffer severely from blood-poisoning. Therefore, if there be any doubt as to the efficient condition of the house drainage, no such patient should come within the neighbourhood of the w.c. A similar precaution applies to women after child-birth for very evident reasons.

All soiled clothing and utensils must be removed out of the sick-room at once. Sheets must be changed as soon as soiled, however often the change may be required.

No food, especially no liquid food, must be left in the sick-room. Milk, beef-tea, jelly, etc., become feeding-grounds to nurture up generations of poisonous organisms, and the patient is subsequently drugged with a concentrated solution of them. No liquid food should be kept in a locker by the patient's bedside. For similar reasons, the drinking water of the sick-room should be frequently changed. Filters should not be allowed in wards, as they may become sources of poisoning on a large scale.

Look upon every case of doubtful disease as infectious until the contrary is decided for you by the medical attendant. Isolate such patients; have no gossiping in the room. To secure this point thoroughly, get the blinds down, allow no talking, no moving about, and the sympathetic visitors will find the place very "damping to their spirits." You will, of course, be thought a very disagreeable person, but kept in remembrance as a good nurse. In infectious cases exclude children, and all useless visitors; allow no kissing, no maudlin sympathy—these exhaust the patient where they do not communicate disease. Use old rags for handkerchiefs, and old sheets, old bedding—in short, everything that can be destroyed completely without the least compunction.

Lastly, a few precautions must be given with regard to your personal health. Contagion, it has been said, has no sense of justice, but in reality its incidence is not quite haphazard. The ignorant and the careless are attacked more often than those who, besides trusting in Providence, take all precautions which reason and experience suggest as useful.

You must invariably wash your hands before touching any food or eating any food. To eat with unwashen hands may not defile a man, but it certainly defileth a nurse very seriously.

Take care of cuts, scratches, punctures, agnatils, and spines about your hands. The skin-folds covering the roots of the nails must not be interfered with in the least. The nails may be cut short, but not to the quick. Take especial precaution if attending patients with irritating discharges, sore eyes in the newborn, etc.

If nursing a severe throat case, it would be well to gargle your throat from time to time.

If you feel out of sorts when attending an infectious case, report the fact at once, as you may have the misfortune to infect several people unwittingly, before you are down yourself.

